# Virotherapy in Germany—Recent Activities in Virus Engineering, Preclinical Development, and Clinical Studies

**DOI:** 10.3390/v13081420

**Published:** 2021-07-21

**Authors:** Dirk M. Nettelbeck, Mathias F. Leber, Jennifer Altomonte, Assia Angelova, Julia Beil, Susanne Berchtold, Maike Delic, Jürgen Eberle, Anja Ehrhardt, Christine E. Engeland, Henry Fechner, Karsten Geletneky, Katrin Goepfert, Per Sonne Holm, Stefan Kochanek, Florian Kreppel, Lea Krutzke, Florian Kühnel, Karl Sebastian Lang, Antonio Marchini, Markus Moehler, Michael D. Mühlebach, Ulrike Naumann, Roman Nawroth, Jürg Nüesch, Jean Rommelaere, Ulrich M. Lauer, Guy Ungerechts

**Affiliations:** 1 Clinical Cooperation Unit Virotherapy, German Cancer Research Center (DKFZ), Im Neuenheimer Feld 280, 69120 Heidelberg, Germany; mathias.leber@nct-heidelberg.de (M.F.L.); a.angelova@Dkfz.de (A.A.); christine.engeland@nct-heidelberg.de (C.E.E.); J.Rommelaere@Dkfz.de (J.R.); 2 Department of Medical Oncology, National Center for Tumor Diseases and Heidelberg University Hospital, Im Neuenheimer Feld 460, 69120 Heidelberg, Germany; 3 Department of Internal Medicine II, Klinikum rechts der Isar, Technical University of Munich, Ismaningerstr. 22, 81675 Munich, Germany; jennifer.altomonte@tum.de; 4 Virotherapy Center Tübingen (VCT), Department of Medical Oncology and Pneumology, Medical University Hospital, Otfried-Müller-Str. 10, 72076 Tübingen, Germany; julia.beil@klinikum.uni-tuebingen.de (J.B.); Susanne.berchtold@uni-tuebingen.de (S.B.); Ulrich.Lauer@med.uni-tuebingen.de (U.M.L.); 5 German Cancer Consortium (DKTK), German Cancer Research Center (DKFZ), Partner Site Tübingen, Otfried-Müller-Str. 10, 72076 Tübingen, Germany; 6 Department of Internal Medicine, University Medical Center, Johannes Gutenberg University Mainz, Langenbeckstr. 1, 55131 Mainz, Germany; Maike.Delic@unimedizin-mainz.de (M.D.); Katrin.Goepfert@unimedizin-mainz.de (K.G.); Markus.Moehler@unimedizin-mainz.de (M.M.); 7 Department of Dermatology, Venereology and Allergology, Skin Cancer Centre Charité, Charité—Universitätsmedizin Berlin, Charitéplatz 1, 10117 Berlin, Germany; juergen.eberle@charite.de; 8 Virology and Microbiology, Center for Biomedical Research and Education (ZBAF), Faculty of Health, Witten/Herdecke University (UW/H), Stockumer Str. 10, 58453 Witten, Germany; Anja.Ehrhardt@uni-wh.de; 9 Department of Applied Biochemistry, Institute of Biotechnology, Technical University of Berlin, Gustav-Meyer-Allee 25, 13355 Berlin, Germany; henry.fechner@tu-berlin.de; 10 Department of Neurosurgery, Klinikum Darmstadt, Grafenstraße 9, 64283 Darmstadt, Germany; Karsten.Geletneky@mail.klinikum-darmstadt.de; 11 Department of Urology, Rechts der Isar Medical Center, Technical University of Munich, Ismaninger Str. 22, 81675 Munich, Germany; per.holm@tirol-kliniken.at (P.S.H.); roman.nawroth@tum.de (R.N.); 12 Department of Gene Therapy, Ulm University, Helmholtzstraße 8/1, 89081 Ulm, Germany; stefan.kochanek@uni-ulm.de (S.K.); lea.krutzke@uni-ulm.de (L.K.); 13 Chair of Biochemistry and Molecular Medicine, Center for Biomedical Research and Education (ZBAF), Faculty of Health, University Witten/Herdecke (UW/H), Stockumer Str 10, 58453 Witten, Germany; Florian.Kreppel@uni-wh.de; 14 Department of Gastroenterology, Hepatology, and Endocrinology, Hannover Medical School (MHH), Carl-Neuberg-Str.1, 30625 Hannover, Germany; Kuehnel.Florian@mh-hannover.de; 15 Institute of Immunology, Medical Faculty, University of Duisburg-Essen, Hufelandstrasse 55, 45147 Essen, Germany; KarlSebastian.Lang@uk-essen.de; 16 Laboratory of Oncolytic Virus Immuno-Therapeutics (LOVIT), German Cancer Research Center (DKFZ), Im Neuenheimer Feld 280, 69120 Heidelberg, Germany; a.marchini@Dkfz.de; 17 Laboratory of Oncolytic Virus Immuno-Therapeutics (LOVIT), Luxembourg Institute of Health, 84 Val Fleuri, L-1526 Luxembourg, Luxembourg; 18 Division of Veterinary Medicine, Paul-Ehrlich-Institut, Paul-Ehrlich-Str. 51-59, 63225 Langen, Germany; Michael.Muehlebach@pei.de; 19 Hertie Institute for Clinical Brain Research and Center Neurology, Molecular Neurooncology, University of Tübingen, Orfried-Müller-Str. 27, 72076 Tübingen, Germany; ulrike.naumann@uni-tuebingen.de; 20 Division of Virus-Associated Carcinogenesis, German Cancer Research Center, Im Neuenheimer Feld 242, 69120 Heidelberg, Germany; jpf.nuesch@dkfz.de; 21 Cancer Therapeutics Program, Ottawa Hospital Research Institute, 501 Smyth Road, Ottawa, ON K1H 8L6, Canada

**Keywords:** oncolytic virus, virotherapy, research in Germany, virus engineering, virus targeting, therapeutic transgene, immunotherapy, combination therapy, clinical trials

## Abstract

Virotherapy research involves the development, exploration, and application of oncolytic viruses that combine direct killing of cancer cells by viral infection, replication, and spread (oncolysis) with indirect killing by induction of anti-tumor immune responses. Oncolytic viruses can also be engineered to genetically deliver therapeutic proteins for direct or indirect cancer cell killing. In this review—as part of the special edition on “State-of-the-Art Viral Vector Gene Therapy in Germany”—the German community of virotherapists provides an overview of their recent research activities that cover endeavors from screening and engineering viruses as oncolytic cancer therapeutics to their clinical translation in investigator-initiated and sponsored multi-center trials. Preclinical research explores multiple viral platforms, including new isolates, serotypes, or fitness mutants, and pursues unique approaches to engineer them towards increased safety, shielded or targeted delivery, selective or enhanced replication, improved immune activation, delivery of therapeutic proteins or RNA, and redirecting antiviral immunity for cancer cell killing. Moreover, several oncolytic virus-based combination therapies are under investigation. Clinical trials in Germany explore the safety and potency of virotherapeutics based on parvo-, vaccinia, herpes, measles, reo-, adeno-, vesicular stomatitis, and coxsackie viruses, including viruses encoding therapeutic proteins or combinations with immune checkpoint inhibitors. These research advances represent exciting vantage points for future endeavors of the German virotherapy community collectively aimed at the implementation of effective virotherapeutics in clinical oncology.

## 1. Introduction

Virotherapy research covers a continuum of activities, ranging from endeavors for screening and engineering viruses as oncolytic cancer therapeutics, using cutting edge technology to concerted programs for their clinical translation and implementation. Oncolytic viruses (OVs) feature at least two modes of action: (i) direct tumor cell killing by productive infection and viral spread, and (ii) induction of local and systemic anti-tumor immunity by release of tumor antigens in the context of danger- and pathogen-associated molecular patterns, triggering an immunogenic tumor microenvironment [1,2,3,4,5,6,7,8,9,10]. With the rise of immunotherapy in clinical oncology, induction of anti-tumor immunity by viral oncolysis has received increasing attention, with OVs being explored as components of combination regimens that induce primary anti-tumor responses and/or trigger immune cell infiltration of tumors. Virotherapy exploits the diversity of viruses as pharmacophores featuring a wide range of particle and genome sizes, structures and replication modalities [2,9,11]. The defining feature of OVs is a tumor-selective infection or replication. This oncotropism can be achieved by, e.g., the exploitation of defective anti-viral host responses in cancer cells, allowing for the application of interferon-sensitive viruses or virus vaccine strains [6]. Furthermore, OVs have been engineered to target cancer cells, either at the cell entry level, e.g., by fusion of cancer cell-binding ligands to viral surface proteins, or during post-entry replication steps [2,11,12,13]. The latter has been achieved by deletion of viral genes or gene functions required for replication in normal, but not in cancer cells, as well as expression of essential viral genes from tumor-selective promoters, respectively. Many viral pharmacophores were further optimized in terms of therapeutic potency by genome modifications aimed at enhanced cancer cell lysis or anti-tumor immune activation [2,5,9]. A prominent approach is to insert therapeutic transgenes into viral genomes, deriving so-called “armed” OVs [2,14]. Beyond and in addition to that, combination regimens with immuno-, chemo-, radio- and targeted therapies, as well as surgery, are being explored [15,16,17,18,19]. Proof-of-principle has been obtained for both, successful OV development and clinical translation. As such, a plethora of effective OVs have been characterized in preclinical models, and hundreds of clinical studies have been conducted, both exploring the treatment of a wide spectrum of cancer entities and different application routes [20,21]. The demonstration of favorable safety profiles in patients represents a key milestone for the clinical implementation of virotherapy. Furthermore, while most clinical studies have explored intratumoral virus application, OVs have been shown to reach tumors after intravenous application and a durable response of a patient with advanced metastatic disease after a single systemic virus application has been reported [22,23]. The first marketing approval of an OV, T-VEC (Imlygic^®^), was obtained in the US and EU in 2015 [24], establishing virotherapy in routine clinical oncology.

However, formidable challenges remain to be addressed in order to implement OVs as an effective cancer treatment modality for wider use in routine clinical oncology. Foremost, more potent OVs are needed in order to facilitate improved therapeutic outcomes in an increasing range of tumor entities. Limitations that need to be overcome include virus-neutralizing blood factors and virus-sequestering phagocytes [25,26,27], blocks to viral replication and spread in cancer cells and in the tumor microenvironment (TME) [2,6,9], and sub-optimal immune activation or an immunosuppressive TME [1,3,5,7,8,10]. These roadblocks must be addressed to facilitate effective systemic virus application, more potent direct tumor cell killing, and/or immune cell-mediated systemic cancer cell eradication (even after local OV application), respectively. OV combination regimens provide further opportunities, as has been explored so far primarily in prime-boost regimens for OV-mediated tumor vaccination [17]. At the same time, adverse host defenses and side effects need to be kept to a minimum. With the development of more powerful OVs and the manufacturing capabilities to produce and, thus, administer them at higher doses, targeting strategies will be of increasing importance. Of note, emerging virus pharmacophores, a panel of engineering technologies, as well as opportunities for novel combination regimens are available to address these challenges. Finally, more OVs need to progress towards clinical exploration and ultimately marketing approval, a complex process involving various scientific, medical, infrastructural, and regulatory challenges, many of them specific to the OV of choice. While the first approved virus is a herpes virus, it is expected that several virus platforms will provide successful virotherapeutics in the future, depending on the targeted tumor entity, route of application, therapeutic modality (e.g., “arming”), and engineering opportunities.

With this review being part of the special edition on “State-of-the-Art Viral Vector Gene Therapy in Germany”, the German virotherapy community, represented by the authors of this review, provides an overview of German virotherapy research activities. While conceptually different, there is major overlap with gene therapy, which frequently exploits virus-based, replication-deficient gene transfer vectors. Examples include virus engineering approaches towards target cell specificity, understanding and manipulating anti-viral host responses, or process development for virus manufacturing. In fact, some efforts for OV development have evolved from gene therapy research, such as the development of oncolytic adenoviruses, which exploited established approaches for engineering of adenoviral gene transfer vectors. Furthermore, OVs “armed” with therapeutic genes actually represent—from the gene therapists’ perspective—replication-competent gene transfer vectors delivering a therapeutic gene that mediates direct or indirect tumor cell killing. Virotherapy and gene therapy research have always been strongly integrated in Germany and have been active within the German Gene Therapy Society (DG-GT). While the German virotherapy landscape was introduced in a review in 2017 [28], here we present an update with a focus on recent and on-going research activities in pre-clinical and translational virotherapy research.

## 2. Recent Preclinical Virotherapy Research Activities in Germany

**Figure 1:**
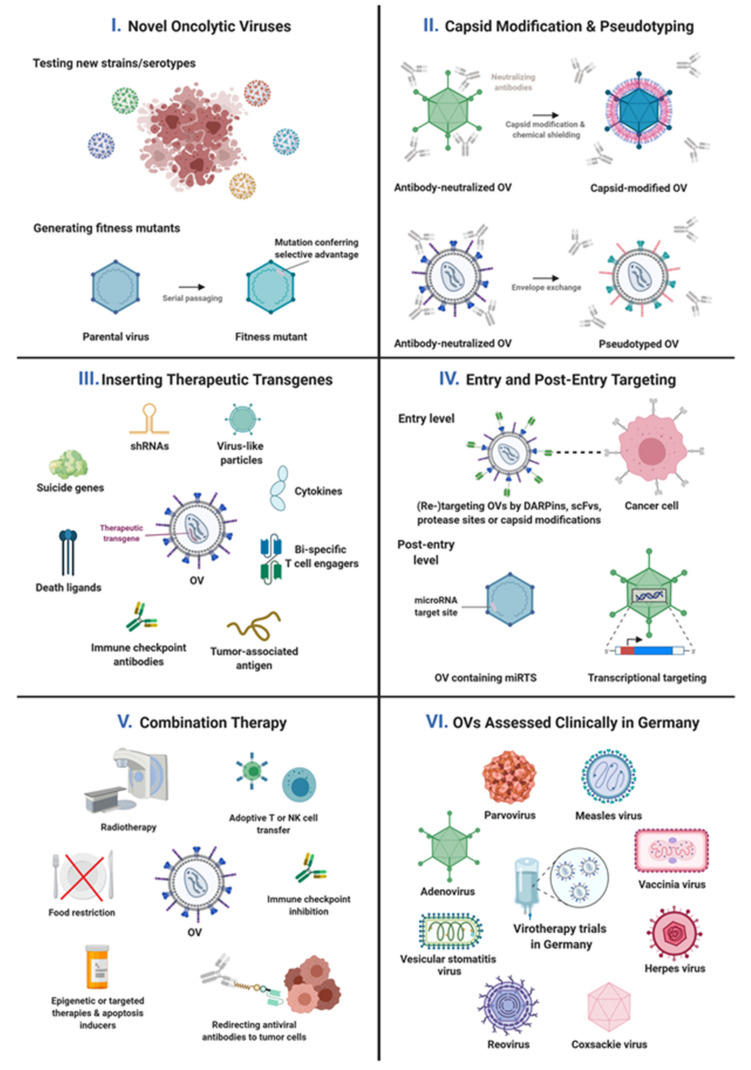
Overview of recent pre-clinical and clinical virotherapy research activities in Germany. Created with BioRender.com.

Exploring novel OVs, advanced OV engineering, and establishing innovative virotherapeutic modalities have been a key stronghold of virotherapy research in Germany [28]. This chapter highlights recent research activities ordered according to the virus platform, i.e., adenovirus, arenavirus, coxsackievirus, herpes simplex virus, measles virus, parvovirus, vaccinia virus, and vesicular stomatitis virus. A comprehensive overview of scientific strategies pursued is provided in Table 1 and Figure 1.

### 2.1. Adenovirus Platform

Oncolytic adenovirus (oAd) engineering and development has been a pillar of German preclinical virotherapy research over the last decades with several groups exploring a spectrum of strategies for developing novel oAds or optimizing them: (i) shielding of oAd particles; (ii) targeting, controlling, or enhancing oAd replication; (iii) “arming” of oAds with therapeutic or imaging genes; (iv) oAd-based immunotherapeutic approaches; and (v) oAd combination treatment (see [28]). In the following, we discuss recent activities in preclinical oAd research in Germany.

The group of Anja Ehrhardt at Witten/Herdecke University focuses on exploring the natural diversity of human adenoviruses (Ads) for the development of oAds. More than 100 distinct human Ads have been identified, but only a limited number of these Ads have been converted into oAds. In a recent study, the group established a genome engineering system enabling cloning of complete Ad DNA genomes from different sources, such as purified virions or infected cells [29]. The technology is based on advanced linear-linear homologous recombination (LLHR) and linear-circular homologous recombination (LCHR). In this initial study, 34 Ad genomes were cloned and tagged with reporter genes. Screening of this reporter-tagged Ad library revealed an Ad-type dependent uptake and oncolysis efficiency in osteosarcoma-derived [29] and breast cancer cell lines [30]. Meanwhile, this cloning system was applied to obtain genetic access to other emerging human Ads [31]. The group is currently adopting interesting candidates into oAds by adding, for instance, tumor-specific promoters driving expression of early virus transcription units and developing novel serotype-derived oAds for targeting of GI cancers (collaboration with the group of Dirk Nettelbeck within the Clinical Cooperation Unit Virotherapy in Heidelberg headed by Guy Ungerechts). In the future, this platform, based on a broad spectrum of different human Ads, may provide the potential to customize OVs to target specific cancer types.

Florian Kreppel’s group at the University Witten/Herdecke continued its work on the modification of Ad vector capsids by covalent chemical, non-covalent chemical, and genetic means (see [28]). The group’s main focus is to improve systemic vector delivery through the blood stream and to understand molecular mechanisms underlying the toxic effects of mistargeted/sequestered Ad vector particles in mice. One central tool of the group is combined genetic and chemical capsid modification that allows for site-specific attachment of ligand or shielding moieties to the virus capsids [32]. This enables studies on the relevance of different capsid positions for shielding by polymer molecules and for targeting by ligands. The group has set up a model to study the in vivo fate of Ad immunocomplexes by chemical decoration of the vector capsid with carbohydrates [33]. This model can serve to analyze the effects of neutralizing and non-neutralizing pre-existing antibodies on the in vivo biodistribution and toxicity. Since Ad vectors based on different Ad serotypes and species are currently used as vectored vaccines against the COVID-19 pandemic (and will be used during the next years), it can be expected that a very large fraction of the global human population will develop neutralizing antibodies and T cells against Ad species and types that were so far considered to be rare. Therefore, it is even more important than before to understand the effects of (cross-) neutralizing anti-Ad antibodies and cross-reactive anti-Ad T cells in order to safely and efficiently use Ads as virotherapeutic agents (for an innovative approach to exploit Ad-neutralizing antibodies for cancer therapy, see next paragraph). In addition, it can be foreseen that effective shielding will become an important tool for oAds. The Kreppel group has contributed to the analysis of novel amphiphilic dendrimers designed to non-covalently interact with the vector surface. Upon systemic delivery through the bloodstream, vectors coated with such amphiphilic dendrimers showed significantly altered vector biodistribution [34]. Overall, these results demonstrate the potency of non-covalent capsid modifications.

The group of Florian Kühnel at MHH in Hannover has previously explored various aspects of oAd engineering and molecular retargeting of oAds using bispecific adapter proteins (see [28]). In contrast to adapter proteins, genetic modification of the viral receptor-binding fiber protein allows for the cross-generational maintenance of the altered target cell tropism during infection. However, options of fiber engineering without compromising the structural integrity of the virus are limited. In collaboration with the group of Rita Gerardy-Schahn (Department Clinical Biochemistry, MHH), a chimeric fiber was designed wherein the knob domain is genetically replaced by the bacteriophage-derived endosialidase EndoNF. This protein combines retargeting properties with essential structural features for a genetic fusion with the adenoviral fiber shaft. The resulting chimeric fiber facilitates a stable molecular retargeting of oAds to infect polysialic acid expressing tumors, such as glioblastoma or small cell lung cancer [35]. In a recent work, the Hannover groups developed an innovative approach to re-direct Ad-neutralizing antibodies to mediate tumor cell killing. Neutralizing antibodies against OVs, either treatment-induced or pre-existing (after childhood infection or vaccination with Ad-vectored vaccines), are a limiting factor of virotherapy because they inhibit effective virus spread. Nevertheless, these neutralizing antibodies represent a potent, yet unexploited immunological resource: Niemann et al. have developed a strategy to convert this undesired immune response against OVs into a tumor-directed immune attack [36]. They generated a tumor-directed adapter molecule which harbors a dominant immunogen of Ad serotype 5, being a frequently used OV. In Ad-immunized mice, intravenous injection of these adapters delayed the growth of syngeneic tumors. The antitumor effect was attributable to CD8^+^ T cells but also required the activity of natural killer (NK) cells. Interestingly, retargeting of preexisting antiviral antibodies also enabled a significant tumor response to PD-1 inhibition. Antibody retargeting after intratumoral virotherapy was highly effective in a murine MC-38 colon cancer and additional application of a PD-1 checkpoint inhibitor resulted in long-term survival of the majority of treated mice. These results confirm that virotherapy and antibody retargeting may promise to activate immunologically ‘cold’ tumors for checkpoint inhibitors.

The group of Stefan Kochanek and Lea Krutzke, with Astrid Kritzinger, Robin Nilson, and Frederik Wienen pursue different projects in the areas of oncolytic virotherapy, genetic vaccination and use of mesenchymal stroma cells (MSCs) as carriers. Therapeutic target with replicating Ads is the head and neck squamous cell carcinoma (HNSCC). In their research, they pay special attention on targeting not only the actual cancer cells but also cells of the tumor microenvironment (TME). In the last years, they have developed a capsid-modified oAd, which shows not only substantially enhanced and Coxsackievirus and adenovirus receptor (CAR)-independent infection of cancer cells, but also significant infection of cells of the TME. The virus is currently being investigated in preclinical tumor animal studies. Prospective studies will analyze if the introduced mutation can also be transferred to other Ad types. Important aspects are also production- and quality-related issues that are currently being addressed. The Kochanek group further investigates the use of MSCs as carrier cells to enable systemic administration of oAds. Allogenic or autogenic MSCs naturally migrate towards tumor sites; hence, represent promising shuttle cells to transport viruses to their site of action while protecting them from cellular or non-cellular sequestration mechanisms en route. The group developed a readily applicable method to substantially enhance the ex vivo virus transduction of infection-refractory MSCs, which, to date represents a major hurdle for the use of MSC as carrier cells in the context of oAds (Nilson et al., submitted). Moreover, they recently established the chicken chorioallantoic membrane (CAM) assay as a quick and low-cost high-throughput tumor model system for the in vivo analysis of systemically or locally injected OVs [37]. The method was already successfully used to investigate tumor targeting capabilities of modified Ads and AAVs [38]; however, it is currently also being evaluated for its applicability to other OVs.

The groups of Henry Fechner at Technical University of Berlin and Jürgen Eberle at Charité-Universitätsmedizin Berlin explore the “arming” of oAds for targeting malignant melanoma. In previous studies, they had constructed AdV-TRAIL, a melanoma-specific oAd with inducible expression of TNF-related apoptosis-inducing ligand (TRAIL) and reported superior therapeutic activity in melanoma cells by TRAIL-induced apoptosis (see [28]). However, melanoma cells characteristically develop resistance to TRAIL [39]. In a recent project, the groups showed that TRAIL resistance can be efficiently overcome in melanoma cells by inhibition of the antiapoptotic Bcl-2 protein Mcl-1. Using siRNAs for transcriptional silencing of five different antiapoptotic Bcl-2 proteins, Mcl-1 was highlighted as the most efficient target to overcome TRAIL resistance [40]. In a follow-up study in cooperation with Florian Kreppel (University Witten/Herdecke) the Berlin groups significantly enhanced AdV-TRAIL cytotoxicity in melanoma cells by Mcl-1 silencing. The effects are the result of enhanced apoptosis and necrosis seen both in TRAIL-resistant and in TRAIL-sensitive melanoma cell lines [41]. The Berlin groups now aim to “arm” AdV-TRAIL with (artificial) microRNAs for Mcl-1 downregulation. In addition, computer-designed Mcl-1 inhibitors [42] shall be used to establish strategies for Mcl-1 silencing and enhancement of the oncolytic activity of TRAIL-“armed” oAds in melanoma.

The groups of Per Sonne Holm and Roman Nawroth at the Technical University of Munich (PSH is now at Medical University Innsbruck), in collaboration with the group of Ulrike Naumann at the Hertie Institute for Clinical Brain Research in Tübingen and the group of Uwe Thiel and Sebastian Schober at Children’s Hospital Schwabing are focusing on further developing their previously established YB-1-dependent oAd XVir-N-31 (see [28]) for treatment of glioblastoma, bladder cancer, and sarcoma. Explored treatment modalities include the combination with targeted therapy approaches, radiation or immune checkpoint inhibition. In this regard, the investigators have recently demonstrated that tumor irradiation, temozolomide or STAT 3/5 inhibitors further strengthened the therapeutic impact of XVir-N-31 in glioma-bearing mice, as well as in bladder cancer [43,44,45], indicating the benefit of combining oAds with established therapies. Currently, the therapeutic effects of XVir-N-31 are evaluated in combination with cyclin-dependent kinase (CDK) 4/6 and bromodomain inhibitors (BETi), both known to affect the cell cycle upon targeting the RB/E2F pathway. Results from the past years indicate strong synergistic effects, and ongoing research is focusing on the molecular basis for the observed strong increase in cell killing. Importantly, the increase in cell killing is accompanied with a further stimulation of the immune response. Furthermore, immune-stimulatory effects of a derivate of XVir-N-31 that expresses a humanized antibody against PD-L1 are being evaluated in experimental glioma using immuno-humanized mice. Initial results support previous findings from other groups, indicating that the immune response against tumor antigens plays a central role in the therapeutic effect of oAd. Based on the results from the past years, and together with the spin-off company XVir Therapeutics GmbH and supported by the German Ministry of Education and Research (BMBF) and German Cancer Aid, clinical phase I/II trials with XVir-N-31 are in preparation for the treatment of glioblastoma and sarcoma.

### 2.2. Arenavirus Platform

Karl Sebastian Lang’s group at the University Hospital Essen has previously developed a new concept for using arenaviruses in immunovirotherapy (see [28]). Work included the lymphocytic choriomeningitis virus (LCMV), which is presently being developed within Abalos Therapeutics GmbH. The overall aim is to maximize the inflammatory signals within the tumor tissue and thereby activate several anti-tumoral immune effector mechanisms within the tumor. One ideal condition for the success of this therapeutic approach is a pre-existing anti-tumoral immunity. To achieve a strong and locally restricted (re-)activation of the immune system the LCMV is used as viral backbone. LCMV is almost non-cytopathic and can persist for several days to months in cell culture or mice [78]. This is one feature, which characterizes LCMV as a strong immune activator [79]. LCMV is hardly neutralized by antibodies and initial control will be achieved by CD8^+^ T cell infiltration in infected organs. The Lang group reported that intravenous application of LCMV into tumor-bearing mice can lead to specific replication of the virus in primary tumors and metastasis for several days [58]. This prolonged locally restricted replication of the virus was correlated with a relatively weak responsiveness to type I interferon (IFN-I). Replication within the tumor led to recruitment and activation of anti-tumoral monocytes, tumor-specific CD8^+^ T cells and NK cells [57,58], thereby resulting in tumor shrinkage. These findings are in line with earlier studies in humans using an LCMV-related virus in patients with different tumors [80]. Six of these patients had a beneficial effect (i.e., an altered course of disease) with a distinct destructive effect on the malignant tissues. This hints at a so far completely ignored opportunity to treat cancer patients with an arenavirus-based therapy. In current studies of the Lang group, an adaptation of the virus to the tumor tissue is achieved by specific selection of tumor-prone virus mutations. This so-called Fast Evolution Platform aims to enhance the efficiency, e.g., via accelerated viral replication, and specificity of the virus for specific tumor types, the latter in order to limit potential side effects. Whether such newly tumor-tropic viruses will enhance the already known anti-tumoral effects of LCMV will have to be explored in more detail in the near future. Abalos Therapeutics develops such an optimized virus candidate for clinical testing.

### 2.3. Coxsackievirus Platform

Coxsackieviruses are a more recent addition to the German OV research portfolio. The group of Henry Fechner at the Technical University of Berlin, in addition to his work on oAds (see above), has investigated the oncolytic potential of coxsackievirus B3 (CVB3), a single stranded RNA virus of the picornavirus family, for treatment of colorectal carcinomas. The group showed that the CVB3 strain PD, which has a unique receptor tropism to N- and 6-O-sulfated heparan sulfate, was more cytotoxic in colorectal cancer cells than other CVB3 viruses, which use the CAR for cell entry. In a xenograft mouse model of colorectal cancer, PD and two other CVB3 strains significantly inhibited tumor growth, but only PD showed a sufficient safety profile [46]. Pancreatitis and myocarditis may represent serious side effects induced by CVB3s. Thus, Fechner’s group applied a microRNA-dependent de-targeting strategy to prevent virus replication in both organs. After evaluation of potential insertion sites within the viral genome [54], target sites of microRNAs specifically expressed in pancreas and heart were inserted into the 3′UTR of the highly pancreato- and cardiotropic CVB3 strain H3. Long-term in vivo investigations after intratumoral virus injection into subcutaneously established colorectal tumors in nude mice confirmed that H3 replication was completely prevented in pancreas and heart. Importantly, virus replication in tumors remained unaffected, and tumor growth was significantly inhibited [52,53]. Currently, the group is also investigating anti-tumoral immune mechanisms induced by PD, and they aim to increase the oncolytic activity of PD further by tumor cell-specific adaptation of the virus and by insertion of immunomodulatory transgenes into the viral genome [81].

### 2.4. Herpes Simplex Virus Platform

Herpes simplex virus type-1 is a double-stranded DNA virus and is frequently used in oncolytic virotherapy. An important representative of this virus family is T-VEC (Imlygic^®^), the only virus construct approved in the western hemisphere for virotherapy to date, which is characterized by the additional integration of the gene encoding human granulocyte-macrophage colony-stimulating factor (hGM-CSF), intended to trigger an enhancement of the virus-mediated anti-tumor immune response. The group of Ulrich Lauer at the University Hospital Tübingen performed a preclinical assessment of this state-of-the-art OV, using a panel of human neuroendocrine tumor (NET) cell lines. NETs represent a rare and heterogeneous group of tumors originating from the neuroendocrine system and occurring at various anatomic sites, such as the pancreas, lung, and intestine, and their therapy remains a challenge in oncology. It was demonstrated that T-VEC is able to infect human NET cells, already at very low virus concentrations, with a high oncolytic efficiency, to replicate and to subsequently lyse the cells. Moreover, the virostatic drug ganciclovir (GCV) was found to lower viral titers in all cell lines tested and efficiently limit T-VEC-mediated cytotoxicity, representing an important safety feature for future treatments of NET patients [77].

The group of Markus Moehler at the Johannes Gutenberg University Mainz explored the immunostimulatory effects of the T-VEC virus by comparing it with JS-1, which is identical to it except for the hGM-CSF transgene. Moreover, they analyzed the putative synergistic biological and immunological effects of T-VEC with cytotoxic agents in human tumor-immune cell co-culture experiments (Delic and Moehler, manuscript in preparation). They documented increased activation of human CTLs after infection by both HSV-1 strains, as previously reported for H-1PV as well [82]. After human melanoma cell infection with T-VEC or JS-1, human DC maturation was not substantially increased, similar to a previous report for oAd-infected melanoma cells [83].

### 2.5. Measles Vaccine Virus Platform

The exploration of Measles vaccine viruses (MeV) as OVs is another stronghold of German virotherapy research. In previous preclinical work, several teams in Germany addressed various aspects of oncolytic MeV (oMeV) development towards maximum tumor specificity and therapeutic efficacy, i.e., shielding, entry and post-entry targeting, replication control, enhancing oncolytic potency, “arming” with therapeutic genes, potentiating antitumor immunity and combination regimens (see [28]). Here, we report on recent preclinical activities.

The group of Michael Mühlebach at the Paul-Ehrlich-Institut, Langen, has further elaborated on the advantages of using designed ankyrin repeat proteins (DARPins) as targeting domains to direct cell entry of oMeV to tumor-specific surface antigens. Employing these highly affine and stable domains to redirect the tropism of MeV hemagglutinin to EGFR yielded viruses with the same oncolytic potential on receptor-positive tumor cells than non-targeted MeV. Thereby, it became feasible for the group to generate dual-targeted MeV, the entry of which also becomes restricted to a protease-rich environment as found e.g., in glioblastoma multiforme [51]. This approach should be useful especially when applying MeV with higher cytotoxic potential due to arming with a suicide gene, e.g., super cytosine deaminase (SCD see below) [51], since for only EGFR-targeted agents, on-target side-effects have been described, previously. On the other hand, the inherent potential of vaccine-strain derived oMeV to stimulate innate immune pathways and modulate the immunopeptidome as shown in a collaboration spearheaded by the group of Ghazaleh Tabatabai, University Hospital Tübingen [59] has re-enforced the research of the Mühlebach group on analyzing replicating MeV as a tumor vaccine platform. It is a well-established concept that the immunogenic properties of vaccine strain MeV can be readily used to induce immunity against other pathogens by encoding critical antigens of those [84] as the Mühlebach lab recently demonstrated also for SARS-CoV-2 [85]. The group could demonstrate that this vaccine platform technology even breaks tolerance to homologous tumor-associated autoantigens when encoding the unmodified autoantigen or presenting the autoantigen on retroviral virus-like particles (VLPs) [67]. These VLPs are highly immunogenic, per se, but their immunogenicity can be further strengthened by co-display of GM-CSF [86] pointing at further potential for optimization.

Mathias Leber’s group within Guy Ungerechts’ Clinical Cooperation Unit Virotherapy in Heidelberg focuses on genetic engineering strategies to improve the therapeutic index of OVs with a focus on oMeV (as reviewed in [87]). Recently, the team has explored strategies to enhance oMeV safety and efficacy and analyzed oMeV genomic stability. In this context, the Leber group has previously developed a microRNA-based, post-entry restriction system for oMeVs [88,89]. This system is based on the incorporation of target sequences for differentially expressed microRNAs into the MeV genome. In a recent study, this system was systematically analyzed and further optimized [55]. Viruses harboring microRNA target sites in various positions within the MeV genome were generated and the critical importance of the insertion position on the overall efficacy of virus regulation was reported. Furthermore, it was shown that the mechanism of microRNA-mediated virus control is dependent on the actual microRNA sequence, and likely encompasses both, direct cleavage of target sites and translational inhibition. In a second line of research, the Leber team has systematically analyzed the genomic stability of oMeV during continuous serial passaging in tumor and producer cells [68]. This approach was chosen to mimic extended periods of virus replication in a clinical virotherapy setting. Since RNA viruses can quickly adapt to changing environmental pressures by selecting quasispecies with superior fitness based on beneficial genetic alterations, these changes could potentially weaken their safety profile. The distribution of consensus mutations detected after a full year of serial passaging was non-random, indicating different levels of genetic constraints in different regions of the genome. Altogether, the number of consensus mutations detected in the genomes of serially passaged viruses was remarkably small, further underlining the genomic stability and excellent safety profile of oMeV. In a third line of research, the Leber group aimed at combining the priorities of safety and efficacy in a single engineered virus for chemovirotherapy of pancreatic cancer [56]. Here, target sequences for miR-148a, which is downregulated in pancreatic ductal adenocarcinoma (PDAC) but expressed in multiple healthy tissues of the gastrointestinal tract, were inserted along with the prodrug-converting enzyme cytosine deaminase-uracil phosphoribosyl transferase (CD-UPRT here *E.coli*-derived) into the genome of an oMeV. The microRNA target sites restricted replication and spread of the virus in miR-148a-expressing cells, while allowing for unaltered oncolytic efficacy in PDAC cell lines. The prodrug convertase CD-UPRT converts systemically administered, non-toxic 5-FC (5-fluorocytosin) into the chemotherapeutic drug 5-FU (5-fluorouracil), thus allowing for a localized chemovirotherapy. The group could demonstrate superior anti-tumor efficacy of the MeV-CD-UPRT virus in combination with 5-FC both, in vitro and in vivo. Taken together, this approach demonstrated the feasibility to generate dually modified oMeVs for enhanced safety and efficacy. Currently, the Leber team is working on novel small RNA-based engineering technologies as well as on combination therapy approaches including radio-, immuno- and pharmacovirotherapy.

The group of Christine Engeland within the Clinical Cooperation Unit Virotherapy in Heidelberg headed by Guy Ungerechts (C.E.E. is now at Witten/Herdecke University) has a strong focus on MeV for targeted immunomodulation [90,91,92]. This strategy employs the viral vector for delivery of immunomodulators to the tumor site, thereby increasing the therapeutic window. Moreover, immunomodulators may complement anti-tumor immune effects of oncolysis, leading to synergistic effects. Following this approach, bispecific T cell engagers (BiTEs) were introduced into the MeV platform [64]. BiTEs consist of two antibody single-chain variable fragments (scFv) binding a tumor surface antigen and CD3 on T cells, thereby mediating tumor-directed T cell cytotoxicity. BiTEs have demonstrated clinical efficacy against hematological malignancies. However, difficulties with delivery and toxicities have so far hampered broader application, also against solid tumors. The Heidelberg team showed that MeV-encoded BiTEs are functional and recruit endogenous T cells in vivo. MeV BiTE prolonged survival compared to MeV encoding a non-binding BiTE, parental MeV, and BiTE only. Gene expression profiling revealed signatures linked to T cell activation, but also exhaustion, indicating potential for combination with immune checkpoint inhibition. Mice achieving complete tumor remission subsequently rejected tumor re-engraftment, demonstrating induction of durable anti-tumor immunity. Moreover, in patient-derived xenograft models, the combination of MeV BiTE and adoptive immune cell transfer significantly prolonged survival compared to monotherapies. This was the first study to demonstrate efficacy of an OV encoding a tumor-targeting BiTE in both, syngeneic and patient-derived xenograft models, highlighting the potential of this combination [93]. To further improve effector T cell function, Engeland and colleagues had previously demonstrated strong anti-tumor efficacy of an oMeV encoding IL-12, achieving 90% complete tumor remissions in the MC38cea model, a colorectal cancer model in fully immunocompetent C57BL/6 mice [65]. However, analysis of tumor-infiltrating immune cells had indicated induction of activation-induced cell death (AICD) by MeV-encoded IL-12. To prevent AICD, the group designed MeV encoding an IL-15 superagonist [63]. Despite intratumoral T cell and NK cell activation, MeV IL-15 was less effective in both the B16-CD46 and MC38cea tumor models compared to MeV IL-12. This was associated with stronger viral gene expression and immune activation by MeV IL-12, emphasizing the superior efficacy of this MeV construct. Based on these results, clinical translation of MeV IL-12 is now being actively pursued. While BiTEs and cytokines non-specifically activate all lymphocytes, priming and activation of tumor-antigen specific T cells is a key goal in cancer immunotherapy. To this end, MeV harboring epitope cassettes derived from the model antigen ovalbumin and the melanoma antigen trp-2 were generated [66]. In ex vivo co-culture models, these vectors were shown to mediate efficient antigen presentation, priming of naïve and activation of effector CD8^+^ T cells. Vectors encoding secreted epitope variants or epitope strings targeted to the proteasome mediated the strongest IFN-γ responses. This concept can be adapted to diverse heterologous antigens, both cancer-derived (for immunovirotherapy) and pathogen-derived (for vaccination against infectious diseases). Further, MeV vectors can be combined with vectors derived from other virus platforms in prime-boost regimens to enhance antigen-specific immune responses.

Ulrich Lauer’s group at the University Hospital Tübingen not only works on herpes viruses (above) and vaccinia viruses (below), but also has a special focus on MeV. In this regard, the group investigated a combinatorial approach employing oncolytic MeV together with activated human NK cells (or PBMCs) in human sarcoma cell lines. In an earlier preclinical study, the Lauer group had demonstrated that MeV exhibits potent oncolytic activity in pediatric sarcomas [94]. However, since there were also sarcoma cell lines that showed primary resistance to MeV-mediated oncolysis, thoughts turned towards combination therapies. It was shown that a combination of oncolytic MeV-GFP virotherapy and activated NK cells resulted in enhanced oncolysis of human sarcoma cell lines compared with the respective monotherapies. In addition, NK cells were activated upon coculture with MeV-infected A673 sarcoma cells [75]. These results not only support the initiation of clinical trials combining oncolytic virotherapy with NK cell-based immunotherapies, but also provide the rationale for potential triple combinatorial approaches, for instance with immune checkpoint inhibitors. The same viral construct was used by the Lauer team in a study investigating the influence of starvation on the oncolytic efficacy in human colorectal carcinoma (CRC) cells. Since it is known that starvation sensitizes tumor cells to chemotherapy while protecting healthy cells in a process called differential stress resistance, the group of Ulrich Lauer examined whether this phenomenon also applies to OVs. It was shown that long-term low-serum, standard-glucose starvation significantly enhanced the efficacy of oMeV-mediated cell killing of CRC cells, whereas it was diminished in normal colon cells [74]. With regard to the treatment of patients, a personalized starvation-enhanced virotherapy could provide benefits for distinct CRC cancer patients; however, possible contraindications such as cachexia, sarcopenia and malnutrition as well as the individual perseverance must be considered in the decision for this particular therapy. In further work of the Lauer group, a MeV was combined with gemcitabine to achieve an enhanced chemovirotherapy for pancreatic cancer. Gemcitabine is a first-line chemotherapeutic agent widely used as a palliative treatment option for pancreatic cancer patients. Moreover, gemcitabine, just like many other cytostatic drugs, is able to induce senescence in tumor cells, resulting in permanent cell cycle arrest and consequently in maintaining cells in a less malignant/less proliferative state. In a previous study, the group showed that MeV can infect senescent cells, including pancreatic cancer cells, replicate in them, and even lyse them more efficiently than non-senescent cells [95]. The Lauer laboratory therefore investigated whether gemcitabine-induced senescent tumor cells can be oncolyzed more efficiently during chemovirotherapeutic combination therapy. It was shown that different pancreatic cancer cell lines treated with both gemcitabine and MeV were lysed with higher efficacy than those treated with the respective monotherapy. Furthermore, gemcitabine-induced tumor cell senescence was not impaired by MeV [71]. These findings pave the way for a new therapeutic option for patients with advanced pancreatic cancer. Moreover, the group of Ulrich Lauer pursues strategies to integrate suicide genes into the genome of OVs which has been reported to increase oncolytic efficiency through bystander killing. In a study with acute myeloid leukemia (AML) cell lines and primary AML cells the oncolytic efficacy of a MeV construct armed with super cytosine deaminase (MeV-SCD), a yeast-derived CD-UPRT, which catalyzes the conversion of the inactive prodrug 5-FC into the therapeutically active and clinically approved compound 5-FU, was investigated. It was demonstrated that MeV-SCD infected the leukemic blasts and significantly reduced the number and viability of leukemic cells by induction of apoptosis. The conversion of 5-FC to 5-FU was found to further potentiate- this effect [69].

### 2.6. Parvovirus Platform

The development of oncolytic parvoviruses (PVs), the smallest viruses clinically explored as OVs, has been pioneered by the group of Jean Rommelaere at the German Cancer Research Center in Heidelberg. The group has explored rodent PVs, in particular H-1PV, for therapeutic applications with the initial report published in 1982 [96], and the first-in-human clinical virotherapy study initiated in Germany in 2011 at Heidelberg University Hospital (EudraCT 2011-000572-33, [97]). In parallel, previous preclinical research at the German Cancer Research Center focused on enhancing delivery, oncolytic potency and immunostimulation of oncolytic PVs (see [28,98]).

Recent work by the group of Markus Moehler at the Johannes Gutenberg University Mainz, in collaboration with Jean Rommelaere’s group, explored H1-PV to further improve the therapeutic success of immune checkpoint inhibitors. The H-1PV-induced immunogenic cell death was accompanied by increased expression of the immune checkpoint proteins CTLA-4, PD-1, and PD-L1 [60,62]. Nevertheless, H-1PV-infected human melanoma and colorectal cancer cells triggered maturation of human antigen-presenting cells such as dendritic cells (DC). Combining H-1PV with the immune checkpoint inhibitors ipilimumab, tremelimumab or nivolumab strengthened cytokine release during DC maturation [60,62,99].

The Laboratory of Oncolytic Virus Therapeutics (LOVIT) at the German Cancer Research Center in Heidelberg and at the Luxembourg Institute of Health, headed by Antonio Marchini, pursues three main areas of research to improve the anticancer efficacy of oncolytic PVs: (i) the development of novel combinatorial treatments, which combine PVs with other anticancer agents (recently reviewed in [100]); (ii) the generation of novel engineered PVs with improved oncolytic and immunomodulatory activities (recently reviewed in [101]); and (iii) the characterization of H-1PV life cycle in order to identify cellular factors that could serve as biomarkers to predict the response of PV-based treatments or guide the identification of new drugs synergizing with PVs in killing cancer cells [102]. The LOVIT laboratory recently found that sublethal doses of BH3 mimetics, namely ABT-737 and ABT-199, potentiate the anticancer activity of H-1PV by cooperating with H-1PV in inducing immunogenic cell death. The co-treatment triggers major disturbances at the level of mitochondria, lysosomes and the endoplasmic reticulum, and it is associated with oxidative stress and calcium release [72,102]. On a more fundamental level, the Marchini group characterized the entry pathway of H-1PV in cancer cells. First, it was shown that laminins, in particular those containing the laminin γ1 chain, modulate H-1PV cell attachment and entry. Silencing of *LAMC1*, the gene encoding the laminin γ1 chain, strongly decreased H-1PV cell transduction by impairing H-1PV attachment at the cell membrane. In particular, H-1PV binds to sialic acid moieties present in laminins. A direct correlation between H-1PV oncolytic activity and *LAMC1* mRNA levels was found in 59 cancer cell lines from different tumor entities, suggesting that tumors with elevated levels of γ1-containing laminins are more susceptible to H-1PV-based therapies [49]. Second, Marchini’s laboratory found that H-1PV cell internalization occurs via clathrin-mediated endocytosis, a process that is dependent on dynamin. H-1PV traffics from early to late endosomes, with acidic pH being necessary for a productive infection [48]. This study also revealed that siRNA-mediated silencing of caveolin-1 increased H-1PV transduction of cancer cells, suggesting that caveolin-1 is a negative modulator of the H-1PV life cycle. Further studies are required in order to translate this new knowledge into more effective H-1PV-based therapies.

Jürg Nüesch’s group, also at the German Cancer Research Center in Heidelberg, explores PV fitness mutants for improved oncolytic potency. Although H-1PV proved to efficiently infect and kill a variety of tumor cell lines, success of virotherapy may be hampered in certain cancer entities and/or distinct patients. Such a limitation could be due to the restricted tissue tropism of H-1PV and/or its inability to produce progeny viruses and spread through the patient’s neoplastic tissue. To generate propagation-competent H-1PV variants endowed with increased therapeutic impact on brain cancers, the Nüesch group performed serial adaptation of H-1PV in patient-derived human glioblastoma cell lines. This led to the isolation of H-1PV variants characterized by an in-frame deletion in the NS region and 1–3 amino acid substitutions in the capsid surface [47]. To further enlarge the spectrum of oncolytic PVs, ongoing work of the group determines the genome sequences of PV strains derived from different species and originally isolated as contaminants of various human cancer cell lines ([103]). Obtained sequences are currently used to produce replication-competent molecular clones. In addition, diagnostic tools (e.g., mAbs) are prepared to enable assessment of the oncolytic potential of H-1PV and other PV strains in various cancer entities.

The group of Assia Angelova together with Jean Rommelaere, within Guy Ungerechts’ Clinical Cooperation Unit Virotherapy in Heidelberg, currently develops a heterotypic pancreatic ductal adenocarcinoma (PDAC) spheroid system. Spheroids are generated with PDAC cells, fibroblasts and endothelial cells and allow further coculture with immune cells. They offer a relevant preclinical tumor model for analysis of the tumor-suppressive and immunostimulating capacity of oncolytic PVs and other OVs presently in development.

### 2.7. Vaccinia Virus Platform

The group of Ulrich Lauer at the University Hospital Tübingen not only investigates a suicide gene-armed MeV construct (MeV-SCD, see above and clinical research chapter), but also a vaccinia virus (VV) Lister derivative (GLV-1h94) encoding the same prodrug-converting enzyme, which locally converts the prodrug 5-FC into the chemotherapeutic compound 5-FU. In a systematic evaluation of the NCI-60 tumor cell panel using GLV-1h94 as monotherapy, different levels of cellular resistance were observed within the cell lines, which, however, could be completely overcome by activation of the prodrug system. A more detailed study of the prodrug system revealed that the cytotoxic effect of converted 5-FU is directed either against the cells or against the viral particles, and this process apparently relies on the balance between cell line-specific susceptibility to GLV-1h94-induced oncolysis and 5-FU sensitivity [70]. In further work by the Lauer lab, the oncolytic VV derivative GLV-1h68, which the group previously explored in a clinical study for treatment of peritoneal carcinomatosis (see [28]), showed great promise in neuroendocrine tumors (NETs). The Lauer group demonstrated that GLV-1h68, which includes three expression cassettes encoding β-glucuronidase, β-galactosidase and green fluorescent protein (GFP), exhibits stable cytotoxicity in human NET cells of various anatomical origins and also a highly efficient production of progeny virus particles. Moreover, additional combination with the mTOR inhibitor everolimus, which is approved for treatment of metastatic NETs, did not impair replication of GLV-1h68 suggesting that combinatorial treatment is not an obstacle for further development of the approach [73].

Markus Moehler at Johannes Gutenberg University Mainz analyzed the two oncolytic VVs, JX-594-GFP^+^/hGM-CSF (JX-GFP), which is derived from JX-594 [22] and TG6002 [104] which are genetically modified by secreting hGM-CSF or encoding CD-UPRT for converting 5-FC into 5-FU, respectively [61]. In their human melanoma model, they compared the properties to kill human tumor cells and again induce immunogenic cell death (ICD). Combined treatment of JX-GFP or TG6002 with 5-FU resulted in strongly reduced tumor cell viability. TG6002 in combination with 5-FC did not significantly strengthen the reduction of cell viability in this setting. After viral infection, the ICD markers calreticulin and high mobility group 1 protein (HMGB1) and strong DC maturation were detected. Thus, JX-GFP and TG6002 lyse human melanoma cells and induce immunostimulatory effects to promote human antitumor immune responses [61].

### 2.8. Vesicular Stomatitis Virus Platform

Vesicular stomatitis virus (VSV), and its application in virotherapy, has been the focus of research activities of the group of Jennifer Altomonte at the Klinikum rechts der Isar, Technical University of Munich. VSV is a promising candidate for oncolytic virotherapy, due to its broad host cell tropism and rapid and robust replication and tumor cell lysis; however, the clinical translation of VSV has been substantially hindered by concerns surrounding the known neurotoxic side effects associated with this virus [105]. Newcastle disease virus (NDV) offers the aspect of viral spread via syncytia and has also demonstrated a promising safety profile in humans [106]; however, as it is a deadly pathogen in its avian hosts, the use of oncolytic strains of NDV has been severely restricted due to the severe environmental risk it poses. The Altomonte group has recently reported a strategy to exploit the beneficial aspects of these viruses, while eliminating the safety concerns of each [50]. The novel chimeric virus, VSV-NDV, utilizes the rapidly replicating VSV backbone, wherein the targeting glycoprotein of VSV was replaced with the fusogenic envelope proteins of NDV. By further engineering the fusion (F) protein in the recombinant vector, the group was able to achieve extensive tumor-specific syncytia formation, which is known to be a beneficial mechanism of direct oncolysis, as well as a potent inducer of ICD [107,108]. In vivo, intravenous administration of VSV-NDV led to a nearly twofold increase in survival time in mice bearing multifocal, orthotopic HCC, as well as a 1000-fold elevation in the maximum tolerated dose, compared with VSV [50]. Based on these and additional unpublished data, the group is now working towards clinical translation of VSV-NDV in the context of a planned spin-out, Fusix Biotech. In order to further explore the VSV-NDV platform as a potential treatment partner with established cancer immunotherapeutics, Altomonte and colleagues have recently reported the enhancement of adoptive T cell therapy through combination with fusogenic VSV-NDV in an immunocompetent murine model of melanoma [76]. In this study, it was shown that pre-treatment with VSV-NDV allowed for upregulation of MHC-I on tumor cells and enhanced recruitment of adoptively transferred cytotoxic T cells, resulting in synergistic treatment responses. Strikingly, therapeutic responses were not limited to tumors directly injected with VSV-NDV, but abscopal effects in contralateral tumors were evident as well, which resulted in a significant survival prolongation. The group now focuses on establishing an expanded proof-of-concept in more challenging preclinical tumor models, as well as the development of enhanced immunostimulatory VSV-NDV vectors through arming and optimized combination approaches with other cancer immunotherapeutics.

## 3. Recent Clinical Virotherapy Research Activities in Germany

As with most novel therapeutics, their translation from the laboratory into clinical trials and, ultimately, clinical routine, represents a tremendous undertaking often spanning many years, if not decades. This is especially true for completely new classes of drugs (such as OVs) with a potential for previously unknown adverse side effects. As reflected in the study protocols of completed and ongoing early virotherapy trials, considerable emphasis is put on safety aspects including biodistribution and shedding of virotherapeutics. Along the way from bench to bedside, the vast majority of therapeutic candidates drop out and the many reasons for this include lack of efficacy, severe adverse events, regulatory hurdles, manufacturing issues and financial bottlenecks. While most OVs are still being developed pre-clinically or clinically, the first OV therapeutic (Talimogene laherparepvec, T-VEC, trade name: Imlygic^®^) has received approval for late-stage melanoma therapy by the FDA and EMA in 2015 [21]. This is widely regarded as a breakthrough for the whole virotherapy field, opening up the potential for routine use of virotherapeutics in the clinic. While T-VEC has demonstrated safety and efficacy in the respective phase III trial (OPTIM [109]) against malignant melanoma, we strongly believe that clinical testing of additional and potentially improved oncolytics will add to our armamentarium in the fight against cancer.

The Paul Ehrlich Institut (PEI), which is the Federal Higher Authority being responsible for all clinical virotherapy activities in Germany, actively supports the transfer of virotherapy research results to clinical virotherapy trials in cancer patients. Interaction with the PEI works mainly via the institutionalized platform of the German Cancer Consortium, DKTK [110]. Researchers planning a clinical virotherapy trial are supported at the DKTK platform by counseling sessions in partnership with PEI, which are offered already at an early stage in the development of new active substances and therapeutic methods. As part of their partnership, PEI and DKTK have established special consulting formats to promote the initiation of clinical trials in the academic environment. Research-based physicians and scientists at DKTK who are planning a clinical virotherapy trial are supported by free kick-off meetings to answer general questions and by scientific advisory meetings on product-specific issues. An overview of all ongoing virotherapy studies is provided by the DKTK Study Register of the Clinical Communication Platform (dktk.org/ccpstudienregister). Taken together, the close interaction with PEI has worked out to be very helpful and instrumental, especially in the setup and configuration of novel phase I protocol types. Thus, virotherapy in Germany receives continuous and sustained support.

In the following, we will summarize recent clinical trials that were initiated by or involved investigators in Germany (for an overview see Table 2).

### 3.1. H-1 Parvovirus (H-1PV)

The first PV clinical trial, ParvOryx01 (EudraCT 2011-000572-33), performed at Heidelberg University Hospital, demonstrated the excellent safety profile of H-1PV upon both local and systemic administration in glioblastoma patients. In addition, trial-accompanying research provided a first hint of PV treatment-induced enhanced inflammation (“warming up”) in the tumor microenvironment [97]. Further detailed analysis of resected glioblastoma tissues revealed the formation of large intratumoral immune infiltrates composed of activated (CD25^+^, granzyme B- and perforin-expressing) cytotoxic T lymphocytes (CTLs) [111]. Importantly, tumor-infiltrating CTLs were PD-1-negative and only scarcely scattered Treg cells were detected within the infiltrates. Glioblastoma-associated microglia/macrophages similarly displayed an activated phenotype characterized by increased CD68, cathepsin B and iNOS expression. Production of proinflammatory cytokines, in particular interferon-gamma (IFN-γ) and interleukin-2 (IL-2), was also observed in a subset of ParvOryx01 patients’ tumor samples. The above findings provided valuable first-in-man experience and laid the ground for future parvoviro-immunotherapy clinical developments. Among these, one approach, namely combining H-1PV with bevacizumab and checkpoint inhibitors, deserves special consideration based on encouraging data from recent compassionate use programs in recurrent glioblastoma [112]. Partial to complete tumor remission was documented in patients who received the PV in combination with bevacizumab and PD-1 blockade. The response rate was significantly higher than reported in the literature for bevacizumab and nivolumab applied as monotherapy.

Clinical evidence that immune mechanisms underlie PV-mediated tumor suppression also came from the second H-1PV single center trial in Heidelberg, ParvOryx02 (EudraCT 2015-001119-11) in patients with metastatic pancreatic cancer. In this study, virus administration was found to be associated with a favorable clinical outcome in two out of seven patients, with radiologically proven partial response and remarkably long survival. Moreover, the findings of accompanying research confirmed immunological effects of H-1PV on the tumor microenvironment associated with a favorable clinical outcome (manuscript under review). Therapy was very well tolerated without any clinically detectable adverse events, except elevation of C-reactive protein (CRP) in four out of seven patients. No dose-limiting toxicities occurred, accordingly. Viral shedding data attest an excellent safety profile of H-1PV with consistent formation of anti-drug antibodies after virus administration and no subsequent detection of infectious viral particles in body fluids on day 18 or thereafter. Viral tumor homing after intravenous administration could be determined in patients of all dose levels. Altogether, the clinical experience gathered so far provides a strong impetus for further H-1PV-based cancer immunotherapy development (recently reviewed in [98,100]).

### 3.2. Measles Viruses

The Heidelberg Team headed by Guy Ungerechts has engineered multiple transgene-encoding oMeVs for increased therapeutic efficacy (see preclinical research highlights and [28]). One of the lead candidates, an oMeV encoding a secreted form of interleukin 12 [65] (MeV-IL12), is currently being moved into clinical testing. A phase I/II investigator-initiated trial in Heidelberg is scheduled to be launched in 2022. This trial is sponsored by the Heidelberg University Hospital spin-off company CanVirex AG and will assess the immunovirotherapeutic efficacy of MeV-IL12 against multiple refractory solid tumors (basket trial). With multiple patent families, the Heidelberg team along with CanVirex AG is aiming to launch a series of such immunovirotherapy trials over the next years. Importantly, these trials will be accompanied by a comprehensive translational research program to unravel immune signatures associated with response to immunomodulating oMeV using state-of-the-art techniques, including laser capture microdissection and automated microscopy after immunohistochemistry to quantify tumor-infiltrating lymphocyte subpopulations, cytokine and chemokine profiling by multiplex arrays, TCR repertoire sequencing, analysis of humoral and cellular anti-tumor immune responses as well as tumor expression profiling with neoepitope discovery.

As described previously ([28]), the group of Ulrich Lauer is investigating an oMeV armed with the prodrug-converting enzyme SCD (MeV-SCD), which locally converts the prodrug 5-FC into the chemotherapeutic compound 5-FU. A monocenter investigator-initiated trial (IIT) sponsored by University Hospital Tübingen is scheduled by the Lauer team in Tübingen, in which safety and potential efficacy of MeV-SCD plus prodrug 5-FC combined with pembrolizumab is evaluated in patients with stage III/IV non-small cell lung cancer (NSCLC). The current standard of care for NSCLC is the anti-PD-1 immune checkpoint inhibitor (ICI) pembrolizumab, although in some cases with a low objective response rate. Accordingly, there is an urgent need for novel combination treatments that further enhance the antitumoral efficacy of pembrolizumab. This study aims to additionally administer MeV-SCD IT into NSCLC tumor lesions of patients who are under pembrolizumab monotherapy, however with limited response. The analyses accompanying the study will include the characterization of the tumor-specific immune response in blood samples as well as in tumor biopsies, the investigation of viral parameters such as infection, replication and marker gene expression of MeV-SCD, as well as the determination of the conversion rates of 5-FC to cytotoxic 5-FU derivatives. In addition, the antibody-/nanobody-based immuno-imaging (immunoPET) established at the University Hospital Tübingen will be applied in this study in order to guide and predict the efficacy of this combined immunovirotherapeutic approach. Another projected monocenter IIT (sponsor: University Hospital Tübingen) will investigate MeV-SCD in patients with gastrointestinal (GI) tumors. In this study, patients with GI tumors will be treated IT by endoscopic guidance with MeV-SCD alone or in combination with the prodrug 5-FC or the anti-PD-1 checkpoint inhibitor pembrolizumab. Primary objectives are to determine the safety, tolerability and immunogenicity of each treatment regimen.

### 3.3. Vaccinia Virus

Pexastimogene devacirepvec (Pexa-Vec) is a VV-based oncolytic immunotherapy designed to preferentially replicate in and destroy tumor cells while stimulating anti-tumor immunity by expressing GM-CSF. Markus Moehler (Johannes Gutenberg University Mainz) with investigators from multiple German sites (including Hamburg, Heidelberg, and Munich) promoted a randomized, open-label, international phase IIb trial that investigated whether Pexa-Vec improved overall survival (OS, primary endpoint) over Best Supportive Care (BSC) alone in HCC patients who failed sorafenib (TRAVERSE study) [113]. 129 patients were randomized 2:1. Pexa-Vec was given as a single intravenous (IV) infusion followed by up to 5 IT injections. Unfortunately, a high drop-out rate in the control arm (63%) confounded the response-based endpoints. Median OS for the generally well-tolerated Pexa-Vec plus BSC vs. BSC alone was 4.2 and 4.4 months, respectively. However, induction of immune responses to VV antigens and HCC associated antigens were clearly observed in patients by ELISPOT analyses of immune response to VV, β-galactosidase and tumor antigens before (pre-dose) and 6 weeks after treatment (post-dose). Detection of T-cells specific for tumor-associated antigen peptides with detectable increased responses against MAGE-A1 and MAGE-A3, as well as HCV peptides in HCV-positive patients were also documented. Despite a tolerable safety profile and induction of T cell responses, Pexa-Vec did not improve OS as second-line therapy. The true potential of OVs may thus lie in the treatment of patients with earlier disease stages or minimal residual disease, which should be addressed in future studies.

Since 2019, the PHOCUS multi-center phase III clinical trial has been completed (NCT02562755). Multiple German sites (incl. Aachen, Bonn, Dresden, Frankfurt am Main, Hamburg, Hannover, Heidelberg, Mainz, München, Tübingen, Ulm) participated. In this trial, IT-administered Pexa-Vec followed by sorafenib was compared to sorafenib treatment alone in the first-line treatment of patients with advanced hepatocellular carcinoma. The study enrolled a total of 459 patients of which 234 received Pexa-Vec followed by sorafenib and 225 received sorafenib alone. The trial was terminated early after an interim analysis came to the conclusion that the trial was unlikely to meet its primary objective at the initially planned study end. In July 2020, data collection for primary outcome measure was completed, and we are currently awaiting publication of the final trial results.

### 3.4. Herpes Virus

The MASTERKEY-265/KEYNOTE-034 trial (NCT02263508), sponsored by Amgen was a phase Ib/III trial in unresectable late stage IIIB to IVM1c melanoma with talimogene laherparepvec (T-VEC, Imlygic^®^) in combination with pembrolizumab and was launched back in 2014. However, the phase Ib part was conducted in overseas only. Results were extremely promising with no dose-limiting toxicities, a confirmed objective response rate of 62% and a complete response rate of 33%. Patients who responded to combination therapy had increased CD8^+^ T cells, elevated PD-L1 protein expression, as well as IFN-γ gene expression on several cell subsets in tumors after T-VEC treatment [114]. German trial centers (Berlin, Dresden, Erlangen, Essen, Hannover, Heidelberg, Kiel, Leipzig, Mainz, Mannheim, München, Regensburg, Tübingen, Würzburg) participated later in the phase III part of the trial which was stopped for futility after an interim analysis by the Data Monitoring Committee. No new safety signals were observed and results are anticipated to be presented at ESMO 2021.

Besides, T-VEC was evaluated in several other multi-center clinical trials involving German study sites. This list includes early (phase Ib and/or II) trials for treatment of triple-negative breast cancer and colorectal carcinoma with liver metastases (Eudra-CT No: 2015-005480-16; German study sites: Berlin, Bonn, Tübingen), melanoma (Eudra-CT No: 2019-001906-61; Germany study sites: Berlin, Dresden, Hannover, Heidelberg, Regensburg, Tübingen), or non-resectable liver tumors (Eudra-CT No: 2014-005386-67; German study sites: Berlin, Bonn, Reutlingen, Tübingen).

CERPASS (Eudra-CT No: 2018-003964-30; sponsor Replimune): besides T-VEC, another herpes simplex virus type 1, named RP-1, is investigated in a phase II trial in combination with cemiplimab (anti-PD-1 mAb) in patients with advanced cutaneous squamous cell carcinoma (CSCC). RP-1 is a selectively replication competent HSV-1 virus which contains two additional sequences, one for hGM-CSF and one for gibbon ape leukemia virus fusogenic glycoprotein (GALV-GP-R^-^). The expression of GALV-GP-R- causes enhancement of viral spreading through the tumor, triggered by the induction of syncytia formation in infected tumor cells. The immunogenic cell death evoked by this pathway together with the local expression of hGM-CSF and the additional combination with the checkpoint inhibitor cemiplimab is expected to result in a synergistically enhanced anti-tumor immune response, which is intended to lead to an improvement of clinical benefit. The primary objective of this study is to assess the clinical benefit of cemiplimab applied intravenously as monotherapy compared to cemiplimab in combination with intratumorally administered oncolytic RP-1 in patients with advanced CSCC. The study will now be expanded to include study centers in Germany (e.g., Tübingen and other skin cancer centers).

### 3.5. Adenovirus

RADNET (Eudra-CT no: 2014-000614-64): this single-center phase I/IIa clinical study of oAd AdVince was initially launched in 2016 in Sweden, evaluating the safety of repeated infusions of AdVince into the hepatic artery of patients with metastatic neuroendocrine tumors (NETs). By inserting the human chromogranin A (CgA) promoter and the mouse H19 insulator as well as microRNA target sequences in the 3′UTR of E1A, AdVince is designed to replicate specifically in neuroendocrine tumor cells but not to damage any normal hepatocytes. Furthermore, patients receive cyclophosphamide, if tolerated, as a concomitant therapy to transiently suppress antiviral immunity and potentially increase the therapeutic effect of AdVince. The primary aim of this study is to evaluate the safety and the maximum tolerated dose (MTD) of AdVince for patients suffering from advanced NETs with multiple liver metastases refractory to surgical resection. Secondary objectives include the anti-tumor efficacy and the replication profile of AdVince as well as the humoral and cytokine-mediated immune response triggered by this virotherapy. The study is now scheduled to be expanded with an additional study center in Tübingen, Germany, in order to increase the number of participating patients.

START (Eudra-CT No: 2021-000642-18): the START (**S**afety and anti-**T**umor **A**ctivity of PeptiC**R**Ad-1 in **T**reatment of Cancer) study is an open-label, non-randomized, first-in-human phase I trial of PeptiCRAd-1 against multiple solid tumors (melanoma, triple-negative breast cancer and NSCLC). The adenovirus-based oncolytic encodes two additional therapeutic transgenes (CD40L, OXO40L), which will be expressed in tumor cells upon infection to further stimulate the innate and adaptive immune response. Moreover, the virus is coated with NY-ESO-1 and MAGE-A3 peptides to direct the immune system against NY-ESO-1 or MAGE-A3-positive tumor cells. Patients will be pre-treated with intravenous cyclophosphamide to enhance the therapeutic efficacy of PeptiCRAd-1, which will be administered intratumorally in six individual doses. As a common theme with other OV trials, PeptiCRAd-1 is combined with immune checkpoint inhibition (pembrolizumab). The bicentric trial will be performed at study centers in Frankfurt and Heidelberg (sponsor: VALO Therapeutics).

### 3.6. Coxsackievirus A21

V937 (NCT04521621): a phase Ib/II clinical study of intratumoral administration of V937 (Coxsackievirus A21) in combination with pembrolizumab (MK-3475) in patients with advanced/metastatic solid tumors. Safety and dose finding of the above-mentioned combination is a primary objective, and again, synergistic effects of checkpoint inhibition and oncolytic agent are anticipated (sponsor: MSD; participating trial centers in Germany are Tübingen and Heidelberg).

### 3.7. Reovirus

GOBLET (Eudra-CT No: 2020-003996-16): a phase I/II multiple-indication biomarker, safety, and efficacy study in advanced or metastatic Gastrointestinal cancers explOring treatment comBinations with peLarEorep and aTezolizumab. In this study, the hypothesis is that treatment with pelareorep will prime the TME for checkpoint blockade therapy, thereby increasing PD-L1 expression and the number of new T cell clones within the tumor, both of which are associated with increased response to checkpoint blockade [115]. In this trial, the virus will be administered intravenously. The trial is sponsored by Oncolytics Biotech Inc. and organized by the AIO-Studien-gGmbH (multiple trial centers in Germany).

### 3.8. Vesicular Stomatitis Virus

A phase I open-label, dose escalation trial is planned to investigate a novel replication-competent vesicular stomatitis virus pseudotyped with the glycoprotein of the lymphocytic choriomeningitis virus (VSV-GP) as monotherapy and in combination with an anti-PD-1 mAb in patients with advanced, metastatic or relapsed/recurrent malignant solid tumors. This trial will evaluate the safety and tolerability of VSV-GP when given via intravenous and/or intratumoral routes. Furthermore, early efficacy signals and the MTD or recommended phase II dose for VSV-GP both as monotherapy and in combination with an anti-PD-1 mAb are major trial objectives. This study is sponsored by Boehringer Ingelheim Ltd. with Ulrich Lauer from University Hospital Tübingen as the European coordinating investigator.

## 4. Perspectives

Four years ago, we—the German virotherapy community—concluded in our state-of-the art review in 2017 ([28]) that “successful translation of German preclinical activities has the potential to inspire a boom in early clinical trials in the near future”. In this context, connecting (i) academic research, (ii) technology transfer, and (iii) regulatory processes was identified to be most critical. We do believe that significant advances within all three sectors have been made.

The recent progress in preclinical, translational and clinical virotherapy research in Germany reported in this state-of-the-art review establishes a vantage point for future endeavors that aim at defining new and clinically effective virotherapeutics based on established technology and advancing clinical development of established OVs. As such, it will be of interest to further engineer and develop the emerging new virus strains, serotypes, fitness mutants and chimeras for virotherapeutic applications. Furthermore, recent scientific insights on virus entry, host-virus interactions after systemic virus application and virus biodistribution should be exploited to further improve OVs or optimize application modalities. We are curious to see whether the reported virus targeting approaches will come to wider application when developing OVs with improved efficiency towards clinical application. In this regard, various efficacy-enhanced OVs have become available resulting from enhanced host cell lysis, improved immune activation, and/or encoded therapeutic proteins or RNAs. In the context of immuno-oncology, it will be of interest to investigate how the discussed new virus platforms (e.g., arenaviruses), genetically delivered therapeutic molecules or tumor antigens, combination regimens, or innovative approaches, such as the redirection of antiviral antibodies for cancer cell killing unfold therapeutic potential as combination immuno-(viro-)therapeutics. Increasingly, the conduction of clinical trials will give us the opportunity for systematic reverse-translational activities, which means new assignments for our preclinical research programs.

Regarding the current limitations of and future challenges for clinical trials in the virotherapy field, from the German perspective we believe that safety and feasibility has been demonstrated in the last decade, and thus the focus should be shifted to oncolytic potency. Virotherapists could learn from the brave investigators translating cellular therapeutics, such as CAR T cells, who have been managing severe side effects for years within a specific clinical trial framework. Besides the virus engineering approaches discussed above, future virotherapy trial protocols should include escalated virus doses and multi-modal treatment regimens balancing the thus-far promising safety data with the need for enhanced therapeutic efficacy. To ensure maximum patient safety, these trials should be conducted in specialized centers only, which can provide the necessary infrastructure and trained personnel at all times. Further, we believe that concerted efforts need to be undertaken to select the patients most likely benefitting from virotherapy. This should include the exploration of omics-data to identify and validate robust prognostic (bio-)markers of virotherapy response.

In Germany, a decent number of spin-off biotech companies dedicated to OV development launched within the last couple of years including Abalos Therapeutics GmbH (LCMV), CanVirex AG (MeV-IL12), Oryx (H-1PV), and XVir (Ad XVir-N-31). Internationally, in a growing immuno-oncology market big pharma/biotech acquired several virotherapy spin-offs with upfront payments of USD 300 million and more. If the focus shifted to the above-mentioned German spin-offs, a booster of translational activities can be anticipated. Accordingly, if big pharma companies keep consistent engagement, we can expect a growing number of pivotal multicenter trials.

In terms of harmonization of regulatory processes, we recognized over the last few years that there is an enhanced interaction and fruitful early dialog between the authorities and the research community. Obviously, speed and shape of regulation is triggered by the medical need, which is impressively demonstrated by EMA’s fast-track approvals of (vector-based) vaccines against COVID-19. In oncology, this could be a future blueprint for accelerated evaluation and assessment for certain cancer entities and disease stages.

Altogether, the German OV field has clearly advanced on all relevant levels, including pre-clinical vector development and translational efforts. However, the ultimate benchmark for our success needs to be the clinical benefit of virotherapy for cancer patients. This can only be achieved on a regular basis and in a sustainable manner if we accomplish marketing approval of several OVs for multiple tumor entities in the future.

## Figures and Tables

**Table 1:** Recent preclinical virotherapy research activities in Germany according to scientific strategies.

Scientific Strategy	Description of Research Approach	Virus	Refs
Identifying new viruses as OVs	Screening and/or cloning of virus strains, serotypes, or mutants	adenovirus coxsackievirus parvovirus	[29,30,31] [46] [47]
Shielding virus particles from blood factors or from cellular sequestration	Combining genetic and chemical capsid engineering or exploiting adapter molecules for shielding and targeting of virus particles and for exploration of host interactions	adenovirus	[33,34]
	Exploring carrier cells to enable systemic administration of OVs	adenovirus	on-going work
Exploring/Targeting/Enhancing efficiency of OV cell binding and entry	Unraveling the virus cell entry pathway	parvovirus	[48,49]
	Genetically replacing the cell-binding domain of a viral capsid protein with a tumor-specific ligand	adenovirus	[35]
	Genetic engineering of virus capsid for enhanced entry into tumor cells, cells of the TME, and carrier cells	adenovirus	Nilson et al., submitted, on-going work
	Replacing OV glycoproteins by those of other viruses	VSV	[50]
	Genetic engineering of viral glycoproteins using highly stable and affine targeting domains and selected protease recognition motifs for combined receptor and protease targeting	measles virus	[51]
	Combining genetic and chemical capsid engineering or exploiting adapter molecules for shielding and targeting of virus particles and exploration of host interactions	adenovirus	[33,34]
Post-entry targeting of OV replication	Expression of essential viral genes from tumor-selective promoters	adenovirus	on-going work
	Insertion of microRNA target sites into viral genes for mRNA destruction and/or translational inhibition in healthy tissues	coxsackievirus measles virus	[52,53,54] [55,56]
Enhancing oncolytic activity or tumor-specificity of OVs	Enhancing oncolytic activity of OVs: production of fitness mutants with enhanced oncosuppressive capacity	coxsackievirus parvovirus	on-going work [47]
	Enhancing the tumor-specificity of OVs by selecting mutated viruses in a fast evolution platform	arenavirus	on-going work
Immune effects of OVs and enhancing their immuno-stimulatory potency	OV-induced activation of innate and (anti-tumor) adaptive immunity	arenavirus measles virus parvovirus vaccinia virus	[57,58] [59] [60] [61,62]
	Enabling OV-induced syncytia formation as immunogenic cell death by replacing viral glycoproteins with heterologous fusogenic envelope proteins	VSV	[50]
	Expression of immunomodulators (ICIs, bispecifics, cytokines)	adenovirus coxsackievirus measles virus	on-going work on-going work [63,64,65]
	Expression (and presentation) of tumor antigens for genetic vaccination	measles virus	[66,67]
OV stability	Analysis of genomic stability of OVs	measles virus	[68]
Expression of therapeutic proteins or shRNAs by OVs	Induction of apoptosis by expression of death ligands or RNAi-mediated inhibition of anti-apoptotic proteins of intrinsic apoptosis pathways	adenovirus	[41] and on-going work
	Insertion of suicide genes into the virus genome for genetic prodrug activation	measles virus vaccinia virus	[51,56,69] [61,70]
	Expression of immunomodulators (ICIs, bispecifics, cytokines)	adenovirus coxsackievirus measles virus	on-going work on-going work [63,64,65]
	Expression (and presentation) of tumor antigens for genetic vaccination	measles virus	[66,67]
Combination therapy with OVs	Combination therapy with radiotherapy	adenovirus measles virus	[43] on-going work
	Combination therapy with chemotherapy	measles virus vaccinia virus	[71] [61]
	Combination therapy with apoptosis induction	adenovirus parvovirus	[41] and on-going work [72]
	Combination with targeted therapy	adenovirus measles virus vaccinia virus	[44] and on-going work on-going work [73]
	Combination with epigenetic therapy	adenovirus	on-going work
	Combination therapy with starvation	measles virus	[74]
	Combination therapy with ICI	adenovirus parvovirus	[36] [60,62]
	Combination therapy with adoptive T cell or NK cell transfer	measles virus VSV	[75] [76]
	Combination therapy with anti-viral antibody-retargeting via recombinant adapters	adenovirus	[36]
Control of OV replication (safety measure)	OV inhibition by virostatic drugs	herpes virus	[77]

**Table 2:** Recent virotherapy trials initiated by or involving the authors of this article. Pexa-Vec = Pexastimogene devacirepvec; T-Vec = Talimogene laherparepvec; SCD = super cytosine deaminase; GALV-GP-R^-^ = gibbon ape leukemia virus glycoprotein; ICI = immune checkpoint inhibition; CPA = cyclophosphamide; GBM = glioblastoma multiforme; PDAC = pancreatic ductal adenocarcinoma; GI = gastrointestinal; NSCLC = non-small cell lung cancer; HCC = hepatocellular carcinoma; TNBC = triple-negative breast cancer; CRC = colorectal carcinoma; CSCC = Cutaneous Squamous Cell Carcinoma; NET = neuroendocrine tumors; C = completed; P = planned; T = terminated; O = ongoing.

Virus Platform	Virus Name	Transgene	Combined With	Name	Identifier	Entity	Phase	Status
PV	ParvOryx/ H1-PV			ParvOryx01	Eudra-CT 2011-000572-33	GBM	I/IIa	C
	ParvOryx/ H1-PV			ParvOryx02	Eudra-CT 2015-001119-11	Metastatic PDAC	II	C
MeV	MeV-IL12	IL-12		CanVirex01		GI basket trial	I/II	P
	MeV-SCD	SCD	5-FC + ICI			NSCLC		P
	MeV-SCD	SCD	5-FC + ICI			GI basket trial		P
VV	Pexa-Vec/ JX-594	GM-CSF		TRAVERSE	NCT01387555	HCC	IIb	C
	Pexa-Vec/ JX-594	GM-CSF	sorafenib	PHOCUS	NCT02562755	HCC	III	T
HSV	T-Vec/ Imlygic	GM-CSF	ICI	MASTERKEY-265	NCT02263508	Melanoma	Ib/III	T
	T-Vec/ Imlygic	GM-CSF	ICI		Eudra-CT 2015-005480-16	TNBC and CRC with liver metastases	Ib	O
	T-Vec/ Imlygic	GM-CSF	ICI		Eudra-CT 2019-001906-61	Melanoma	II	O
	T-Vec/ Imlygic	GM-CSF	ICI		Eudra-CT 2014-005386-67	HCC & non-HCC liver metastases	Ib/II	O
	RP-1	GM-CSF, GALV-GP-R-	ICI	CERPASS	Eudra-CT 2018-003964-30	CSCC	II	O
AdV	AdVince		CPA	RADNET	Eudra-CT 2014-000614-64	NET with liver metastases	I/IIa	O
	PeptiCRAd-1	CD40L, OX40L	ICI	START	Eudra-CT 2021-000642-18	Basket trial	I	P
CoxV	V937/CVA21		ICI		NCT04521621	Basket trial	Ib/II	O
ReoV	Pelareorep		ICI	GOBLET	Eudra-CT 2020-003996-16	GI basket trial	I/II	P
VSV	VSV-GP	GP of LCMV	ICI			Basket trial	I	P

## Data Availability

Not applicable.
